# Companion guide to the revised technical guidelines for reliable dc measurements of the quantized Hall resistance for graphene-based devices

**DOI:** 10.1088/1681-7575/ae6c09

**Published:** 2026

**Authors:** Consultative Committee for Electricity and Magnetism, Task Group on Graphene Quantum Hall Resistance Standards, Albert F Rigosi, Hansjörg Scherer, Mathieu Taupin, Luca Callegaro, Hans He, Susmit Kumar, Ali Eichenberger, Mattias Kruskopf, Yanfei Yang, Martina Marzano, Stephen Giblin, Sergiy Rozhko, Takehiko Oe, Dong-Hun Chae, Pierre Gournay

**Affiliations:** 1 Bureau International des Poids et Mesures (BIPM), Pavillon de Breteuil, F-92312 Sevres Cedex, France; 2 National Institute of Standards and Technology (NIST), Gaithersburg, MD 20899, United States of America; 3 Physikalisch-Technische Bundesanstalt (PTB), Bundesallee 100, 38116 Braunschweig, Germany; 4 Laboratoire National de Métrologie et d’Essais (LNE), 29 Avenue Roger Hennequin, 78197 Trappes, France; 5 Istituto Nazionale di Ricerca Metrologica (INRIM), Torino 10135, Italy; 6 RISE Research Institutes of Sweden, Box 857, S-50115 Borås, Sweden; 7 Justervesenet (JV), NO-2007 Kjeller, Norway; 8 Eidgenössisches Institut für Metrologie (METAS), CH-3003 Bern-Wabern, Switzerland; 9 National Physical Laboratory (NPL), Teddington TW11 0LW, United Kingdom; 10 National Metrology Institute of Japan (NMIJ), National Institute of Advanced Industrial Science and Technology, AIST Tsukuba Central 3, 1-1-1 Umezono, Tsukuba, Ibaraki 305-8563, Japan; 11 Korea Research Institute of Standards and Science (KRISS), Daejeon 34113, Republic of Korea

## Abstract

This companion guide revises and expands upon the established technical guidelines for reliable direct current measurements of the quantized Hall resistance, adapting them to the unique characteristics of epitaxial graphene. Graphene has emerged as a viable alternative to traditional Gallium Arsenide heterostructures for metrological applications due to its relaxed operating conditions. These less demanding requirements facilitate the use of low-cost, compact cryomagnet platforms, which is expected to broaden the deployment of primary resistance standards to National Metrology Institutes and other laboratories with limited resources. The guide explores the specific challenges and considerations of epitaxial graphene devices, including aspects of device choice, cooling and handling, contact resistance, conditions of quantization, and general measurements of the quantized Hall resistance. The presented details should assist those seeking to conduct rigorous characterization procedures to verify device integrity, ultimately contributing to a global effort to formally accept graphene-based devices as reliable primary resistance standards.

## 1. Introduction

The quantized Hall resistance (QHR) is a cornerstone of electrical metrology, providing a highly accurate and stable resistance standard [1]. While the foundational journal article entitled “Revised technical guidelines for reliable dc measurements of the quantized Hall resistance” has been instrumental in establishing best practices [2], it primarily focuses on measurements in Gallium Arsenide (GaAs) heterostructures. Since its discovery in 2004, the two-dimensional material graphene has emerged as a viable alternative to GaAs for metrological quantum Hall effect (QHE) applications. This document is intended to be formal extension to Ref. [2] and contains updated information specific to graphene.

A number of studies have shown the universality of the QHE in graphene and GaAs within relative uncertainties ≤ 1×10^−9^, and more recent studies have demonstrated and reviewed the stable operation of practical graphene QHR standards over timescales of several years [3–6]. The calibration of secondary standard resistors in terms of the QHE with relative uncertainties at or below the few-nΩ/Ω level has been conclusively demonstrated in the recent key comparison BIPM.EM-K12 [3]. This document acknowledges that the K12 comparison is currently ongoing and its published data is from a limited number of participating National Metrology Institutes (NMIs). Furthermore, the document’s recommendations may continue to be updated through other media as the comparison progresses.

Graphene is an attractive alternative to traditional GaAs-based devices because of the relaxed conditions required for quantization; temperatures of around 4 K, and magnetic flux densities around 5 T, compared to 1.5 K and 10 T for typical GaAs samples. These relaxed conditions open the possibility of using low-cost, compact cryomagnet platforms, bringing primary standards within reach of NMIs and other laboratories that previously did not have the resources to operate one, and allowing, for example, year-round operation of the primary standard instead of occasional operation for calibrating secondary standards. It is anticipated that in coming years, a number of NMIs that currently operate GaAs QHR standards will switch to graphene, and in addition, laboratories with no previous QHE experience will intend to set up and operate graphene-based QHR standards.

This guide primarily serves as a practical companion to the original guidelines [2, 7], specifically tailoring and expanding upon their principles to address the unique characteristics of epitaxial graphene (EG). The specific challenges and considerations associated with measuring the QHR in this two-dimensional material will be explored, including aspects like device choice, cooling and handling, contact resistance, conditions of quantization, measurement of the QHR and their consistency, and measuring equipment. By providing clear explanations, illustrative examples, and readily applicable techniques including an extended measurement procedure for the device characterization using high-accuracy resistance bridges, this resource assists researchers and metrologists to confidently perform QHR measurements in graphene, contributing to the advancement of precision metrology and the broader application of this remarkable material [1, 8].

Despite the technological maturity of graphene-based QHR devices, a gap remains in the formal acceptance of graphene as a primary standard. This deficiency partly stems from a lack of widely adopted, comprehensive, and standardized measurement protocols specifically optimized for the unique environmental requirements of epitaxial graphene. Therefore, this user-friendly guide also aims to address the need to guide communities with and without formal QHE experience, offering practical insights, sampled instructions, and relevant references for implementing reliable dc measurements of the QHR in graphene-based devices.

To ensure the effective application details in this document, a specific technical baseline is assumed for the reader. Users should possess a fundamental understanding of four-terminal resistance measurements and be familiar with basic cryogenic operations required for low-temperature experiments. For researchers and metrologists entering the field without a formal background in the quantum Hall effect (QHE), it is highly recommended to consult preparatory references such as the seminal work by von Klitzing (1980) [1] and the original technical guidelines by Delahaye (1989) [7] to establish the necessary theoretical and practical foundation.

## 2. Device choice

Epitaxial graphene is highly advantageous for electrical metrology because it offers reliable and scalable access to the QHR at higher temperatures and lower magnetic flux densities compared to traditional GaAs heterostructures.

**Figure 1.**
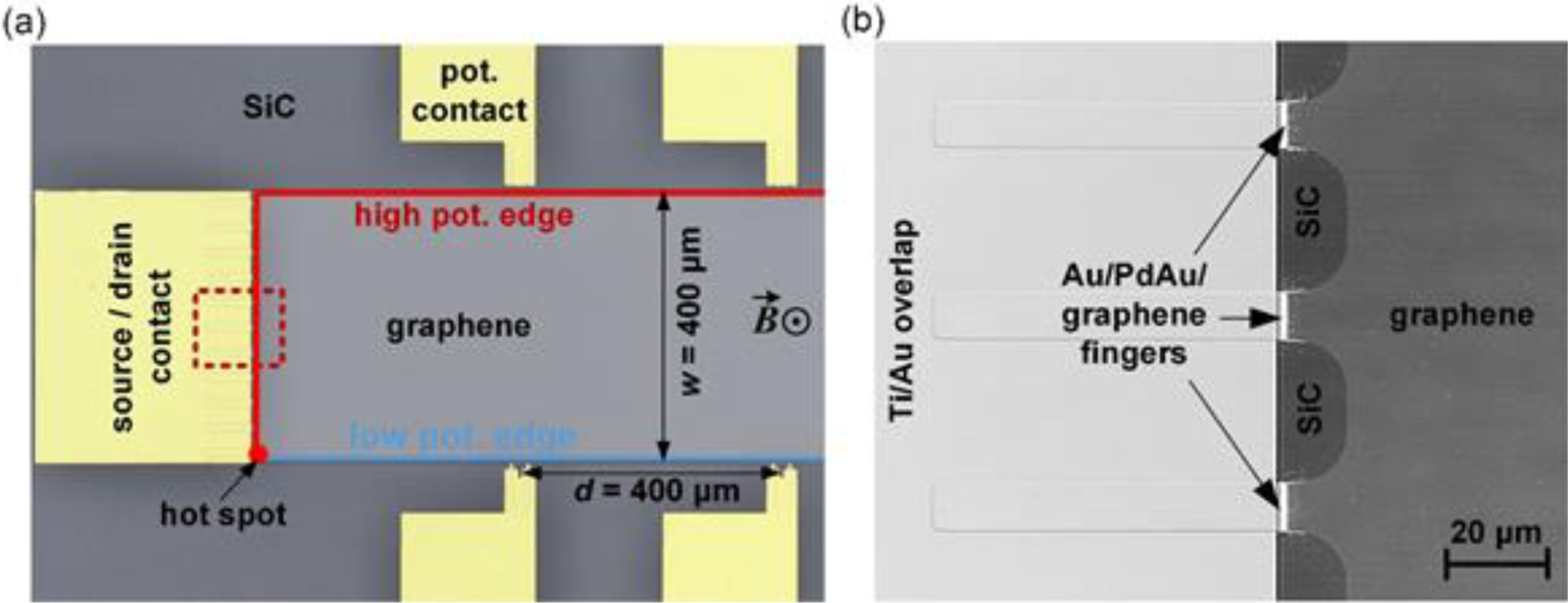
(a) Example of an established graphene quantum Hall resistance (QHR) device with source/drain and potential contacts split into multiple branches. The Hall-bar channel has width *w*, and the distance *d* between neighboring potential contacts is chosen such that *w*/*d* = 1. The dashed-line square in the region of the source/drain contact is shown in (b). In the quantum Hall regime, the current is injected predominantly through the first branch, where a hot spot forms, while successive branches carry only a small fraction of the current. (b) The magnification of the contact region shows the normal-metal Ti/Au bond pad area that overlaps the individual Au/PdAu-covered graphene fingers. This layout effectively implements a multiple-series connection within a single macroscopic contact, thereby minimizing the overall contact resistance.

Typical devices use a Hall bar geometry (rectangular) and have at least six contacts – one pair for current bias, one pair for the transverse Hall voltage, and one pair for longitudinal voltage. Similarly to GaAs-based devices, in the quantum Hall regime, the current in graphene-based devices predominantly enters through the first point in the electrical contact forming a hot spot (see Figure 1 (a)). The size of the Hall bar, and therefore the distance between electrical contacts, should be large enough so that the influence of heating at the hot spots is minimized [9]. If the spacing between the contacts, such as the current injection point and a voltage probe, is too small, the quantum edge states crucial to the QHE are unable to reach thermal equilibrium, potentially leading to inaccurate quantization. And such equilibration lengths usually depend on the properties and parameters of the system exhibiting the QHE (e.g. material, substrate, disorder). For EG, more rigorous studies are still needed to precisely determine this length as a function of such parameters. However, for practical intents and purposes, contact spacings above 100 μm are typically used for metrology, though it should be noted that this number is a conservative empirical rule pending more rigorous determination (see Ref. [9] for a theoretical framework).

To facilitate inter-comparability between laboratories and ensure consistent thermal management at high operating currents (>100 μA), a recommended geometry for EG Hall bars should ideally follow a set of ratios to minimize hot-spot-induced errors. A suggested benchmark for this reference geometry defines the spacing between neighboring voltage probes, *d*, relative to the channel width, *w*, such that the ratio *w/d* ≈ 1. Furthermore, the distance between the source/drain contacts and the nearest voltage probes should be maintained as a multiple of the channel width to ensure thermal equilibration of the edge states. The device shown in Figure 1(a) exemplifies this approach, where *w* = *d* = 400 μm and *w/d* = 1. Adhering to these relative design proportions provides a concrete framework for assessing and comparing the performance of diverse Hall bar designs across different laboratories.

The nature of contact resistance at hot spots prevents complete equilibration between the electrical contact (a metal composition) and the edge channel in the EG. To prevent hot spot interference, one may implement a multiple-series connection [10] within a single macroscopic contact so that small fractions of the current enter through successive branches (commonly 3 branches, see Figure 1 (b) and Refs. [10] and [11] for more detailed information), creating an equal potential between the metal contact and the edge channel. This minimizes the overall contact resistance to near zero, depending on the materials used [11].

Though GaAs-based devices were preferred over MOSFETs [2], EG now holds significant advantages over both. The unique band structure of graphene results in Landau level energy spacing that is much larger than in GaAs (see, e.g., [22] and references therein). This fundamental property leads to two major benefits for metrology:

1. Relaxed Operating Conditions – High-precision QHR measurements in EG have been achieved at higher temperatures (≥ 4.2 K) and lower magnetic flux densities (as low as 3.5 T to 4.5 T). This contrasts sharply with the typical requirements for GaAs (*T* ≤ 2.2 K and *B* ≈ 10 T). A secondary benefit comes from the larger resistance plateau accessible at the *i* = 2 Landau level.
2. Higher Operating Currents – EG-based devices can sustain much higher measurement currents (at least one order of magnitude more than GaAs-based devices) while maintaining quantization, which improves the signal-to-noise ratio and measurement precision. This current level is significantly larger than the typical 10 μA – 100 μA maximum currents of GaAs-based devices.

Table 1 exemplifies the relaxation of parameter spaces (typical values) seen in EG-based devices from various sources. The quantities *R*_xx_ and *ρ*_xx_ correspond to the longitudinal resistance and resistivity, respectively.

## 3. Device cooling and handling

To produce graphene-based devices for accessing the QHR, the growth should permit reliable, homogeneous and scalable fabrication of millimeter-sized Hall bar devices. For these reasons, works on the pursuit of measuring the QHR on exfoliated graphene flakes [19–21] was rapidly abandoned to focus on other growth techniques such as epitaxial growth on the Si face of SiC [22–25], propane-hydrogen chemical vapor deposition (CVD) [18, 26, 27], and polymer-assisted sublimation growth (PASG) [14, 28]. To date, there are several methods that may be used for graphene-based standards production; however, for the purposes of this guide, the primary material referred to is EG. The variety of growth techniques for graphene is a strength compared to GaAs QHRs, relieving pressure on suppliers.

### 3.1. Thermal and Temporal Stability

To preserve the dissipationless transport properties in the quantum Hall regime, efficient thermal coupling of the device to the cryogenic system is essential. The device should preferably be immersed in liquid helium or at least operated in an atmosphere of exchange gas surrounding the sample, to provide reliable heat sinking [29]. Operation in high vacuum, in contrast, can result in poorly defined thermal gradients and elevated local temperatures, which may degrade overall QHR device performance. Knowledge of thermal gradients is important to consider when the graphene-based QHR is realized using cryogen-free systems instead of traditional liquid helium bath cryostats (further discussed in Section 8).

Unlike QHR devices made from GaAs/AlGaAs, where the two-dimensional electron gas is located at an inner interface within a crystal of the heterostructure and well protected from the environment, EG layers are grown on the surface of a substrate material, exposed to the surrounding. Therefore, EG-based QHR devices are highly susceptible to influences of the surrounding environment which can alter their properties. Thus, the temporal stability of such devices is a matter requiring attention.

Recent advances in stabilization of physical properties were achieved, mainly by specific doping methods and additionally by suitable encapsulation techniques which aim at protecting the EG layer from detrimental influences of the environment. These techniques are specific to EG and should be considered before devices are used for metrological purposes.

### 3.2. Techniques for Doping, Adjustment, and Encapsulation

EG used for QHR standards is exceptionally sensitive to surface adsorbates. This sensitivity necessitates precise control over the carrier density through doping, adjustment, and encapsulation. Furthermore, the doping of EG is difficult to control due to high intrinsic doping and Fermi level pinning [30], requiring highly potent doping techniques. Recall that Landau quantization in the quantum Hall regime gives rise to the relation between the carrier density (*n*) and the magnetic flux density (*B*); $n=i\times \frac{\text{eB}}{h}$, where *i* stands for how many Landau levels are filled below the Fermi energy (hence the name ‘filling factor’). Since the carrier density of as-grown EG is typically close to an electron density of 1.0 × 10^13^ cm^−2^, the required *B* is larger than 200 T, not readily available in ordinary laboratories. To observe and measure the QHE under a reasonably small *B*, *p*-doping is required. For instance, the corresponding carrier density to a magnetic flux density of 5 T for *i* = 2 is approximately 2.4 × 10^11^ cm^−2^. The typical charge carrier density that enables reliable operation of the QHR in graphene devices at magnetic flux densities of *B* ≈ 5 T (±1 T) is about 1.0 × 10^11^ cm^−2^ to 2.5 × 10^11^ cm^−2^ [13, 14, 18, 27], where charge carriers can be either electrons or holes. The long-term stability of the electronic transport properties can be improved by controlling the storage conditions, such as the atmospheric gas composition and additional encapsulation techniques [18, 31–33].

#### 3.2.1. On chromium tricarbonyl [Cr(CO)_3_]

As mentioned above, the carrier density in as-grown EG can be highly variable and sensitive to environmental factors, making it potentially unsuitable for high-precision metrology. To overcome this limitation and achieve a stable and controllable carrier density, one may use a technique involving the deposition of Cr(CO)_3_ as a facilitator for controlled *p*-type doping to counterbalance *n*-type doping in EG. [34]. The deposition is performed through thermal decomposition of chromium hexacarbonyl in a vacuum environment. The compound enables a transfer of charge from the EG and, thus, shifts the Fermi level. In the case of Cr(CO)_3_, this interaction generally results in room temperature near-neutral doping. The key to controlling the carrier density lies in regulating the amount of heat applied to the device post-deposition.

As described in [34], one may reversibly tune the carrier density (typically electron density) to a value that is commensurate with the amount of time and heat applied. There are no particular storage requirements for devices treated with this method. Most devices maintain a carrier density close to the Dirac point (so less than 10^11^ cm^−2^), and one example of utilizing this method is to heat such a device to 350 K for about 20 min (in either air or vacuum), typically yielding a density that enables plateau access under 9 T.

Once the device has been heated, it is important to load it into a cryostat or inert gas / vacuum environment within approximately 15 minutes to avoid the device’s natural inclination to return to its steady-state carrier density (a process that typically takes under 24 hours). For a better understanding of the steady-state carrier density timing from which the 15-minute suggestions is derived, see Ref. [34]. This controlled doping allows EG-based devices to exhibit reproducible electrical behaviors, which is essential for their application as a reliable resistance standard, especially when using such devices under equipment constraints, such as a maximum applicable magnetic flux density of 5 T [34, 35].

#### 3.2.2. F4-TCNQ

The molecular dopant F4-TCNQ has emerged as a candidate for a robust doping method, reducing the inherently high electron density in EG and maintaining stable doping levels over extended periods [36]; it has been utilized by several NMIs [12, 14, 31].

The F4-TCNQ molecule provides a strong *p*-doping effect on EG and is volatile under ambient conditions, necessitating stability via an additional polymer matrix, such as poly(methyl-methacrylate) (PMMA). The mixture is deposited on the surface of EG via traditional spin-coating techniques, and varying the mixture composition yields different initial carrier densities [37]. To increase the doping stability over time, a combination of pure polymer spacer layers may be combined with the dopant blend [38] whose recommended lifespan maximum is about 6 months (aging of blends may introduce doping inhomogeneities, thereby degrading quantization accuracy).

The final carrier density after doping can be adjusted by thermal annealing performed under ambient conditions with a simple hot plate (stable up to approximately 80 °C [39]). At higher temperatures, the carrier density becomes increasingly *n*-doped (e.g. the F4-TCNQ loses potency) in an irreversible manner. Precise tuning of the carrier density with annealing necessitates short increments of applied heat combined with monitoring Hall measurements since the mixture properties can vary from batch to batch. This process makes a wide range of achievable carrier densities, most relevantly on the order of 10^11^ cm^−2^ [37].

Once the desired final doping level has been reached, samples can be kept stable over a period of years with careful storage in inert atmospheres, like nitrogen, helium, or vacuum [38]. A simple but effective reported storage method relies on the combination of desiccant and oxygen absorber [18, 31]. Ambient doping tends to reduce the electron density due to hole doping, with noticeable changes occurring within a few weeks [31]. If the doping level/carrier density of a sample has undergone an undesirable change due to ambient exposure, the original value may be recovered by repeating the thermal annealing treatment. This should be performed with caution (i.e. annealing times less than 1 minute) since the effect that the heating has on the molecular dopant is permanent, leading to potentially higher *n*-doping than expected.

This method is rather insensitive to thermal cycling; that is, the same sample can be cooled down and warmed up repeatedly without noticeable degradation or cracking of the polymer stack. Relatively rapid cool-down can be performed without thermal shock impacting the device. Cool-down from room temperature to 2 K (and vice versa) can be performed in less than one hour.

Generally, devices treated with this method exhibit significantly reduced drift in carrier density compared to undoped devices, and they show high temporal stability (sometimes over years) and over multiple thermal cycles [12]. However, the achieved doping level is sensitive to processing parameters during device fabrication [13] and, as mentioned above, to environmental storage conditions such as maintaining low humidity and controlled atmospheric composition to sustain long-term stability [18, 31]. Overall, F4-TCNQ doping provides a robust, stable, and practical doping solution for graphene-based QHR standards, enabling high accuracy and reproducibility [12–14, 31].

#### 3.2.3. On encapsulation and nitric acid vapor

A simple spin-coated polymer layer has not been shown to serve as effective encapsulation owing to the permeation of water and oxygen molecules through the film. One encapsulation method involves the use of a glass lid with a dome at the center, as used in the passivation of organic electronic devices [32]. A second approach instead uses a flip-chip bonded device mounted face-down onto a chip carrier circuit board with pre-patterned contacts, with epoxy applied along the perimeter of the backside of the chip to seal the device [33].

Nitric acid vapor has also been used to introduce strong and effective *p*-doping in EG on the order of 10^13^ cm^−2^. Combined with an annealing process, the carrier density may be adjusted to the desired range. Advantages include reversibility and allowing devices to be stored in ambient environments indefinitely [40]. Disadvantages include the need to repeat treatment after ambient storage, safety hazards in handling nitric acid, and potential damage to electrical contacts.

#### 3.2.4. On annealing and electrostatic discharge

For practical uses of EG-based devices for metrology, both annealing the F4-TCNQ doped device in air and electrostatic discharge may be used to tune the carrier density [41]. This type of annealing is a standard impermanent procedure for fine adjustment of carrier concentration during the fabrication process [36]. The annealing is believed to result in the desorption of F4-TCNQ molecules from the EG - F4-TCNQ interface [39, 42].

An electrostatic corona discharge method in air can be used to reduce the electron density in EG-based devices [41], by the deposition of ions onto a thin polymer layer covering the EG surface. The high electrostatic potential created by the ions results in a reversible gating effect [43]. The doping effect of discharged ions is temporary and will only remain as long as the sample is kept cold. Warming up to room-temperature will quickly recover the initial carrier density.

## 4. Contact resistance

For metrologists utilizing EG-based devices, non-ideal electrical contacts frequently pose significant device-related challenges. Poor contacts are primarily characterized by a high contact resistance (*R*_C_), which can even manifest as non-linear behavior in severe cases.

**Figure 2.**
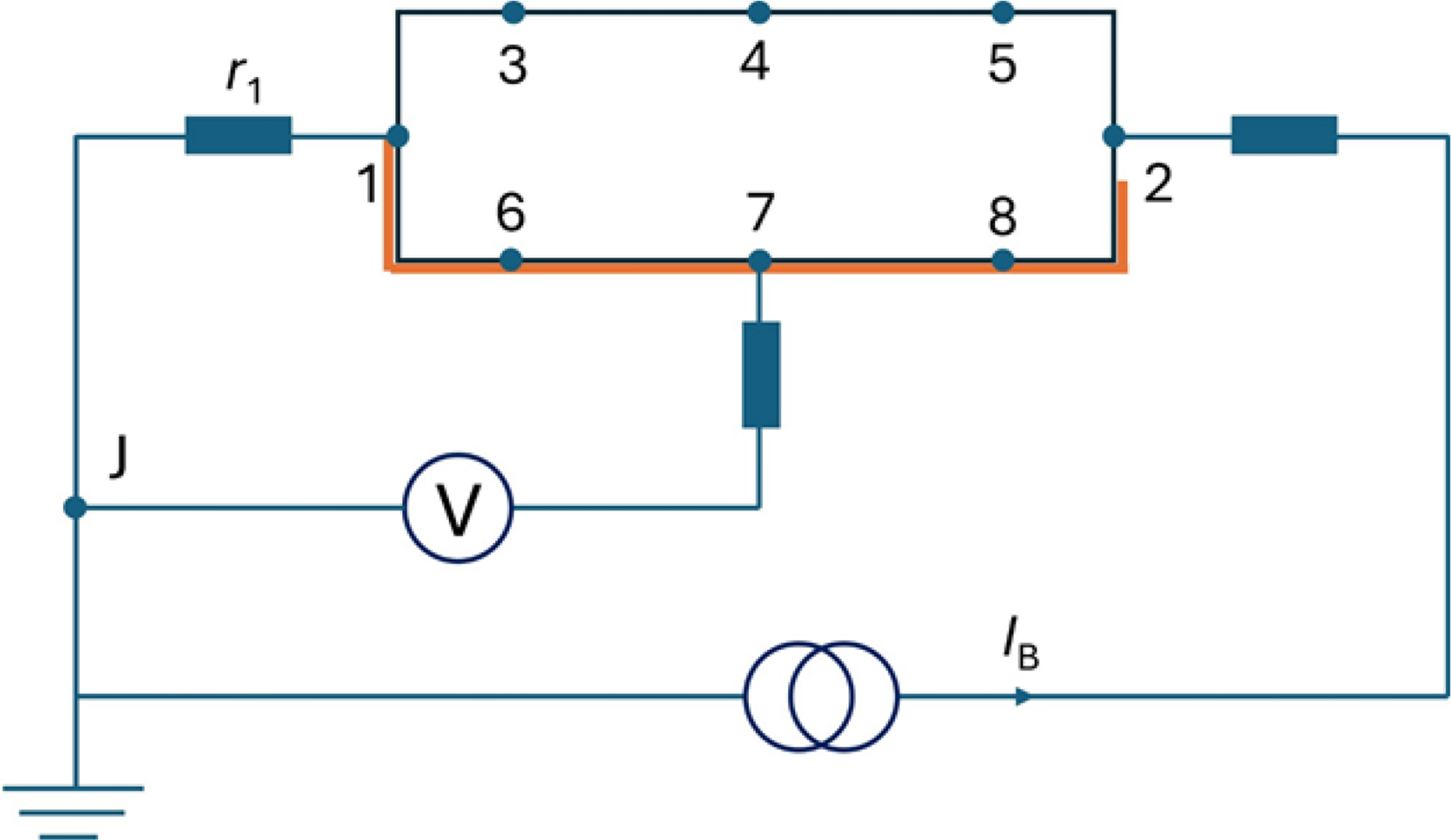
Schematic circuit for three-terminal measurement of contact resistance. The illustrated configuration shows a measurement of *r*_1_, which is the series sum of the resistance of contact 1 and the wiring between the junction point J and the contact. The thick brown line indicates the sides of the sample which are near ground potential. The connections can be permuted to measure all contact resistances, with the constraint that the voltmeter must be connected to a contact on the low potential side of the sample.

Achieving low contact resistance is critical, as any significant resistance can lead to non-equilibrium effects or additional noise, thereby compromising the quantization accuracy [2]. Measurements of the contact resistance in QHR devices are typically performed using three-terminal configurations under zero dissipation conditions (*R*_xx_ ≈ 0). Here, the voltage drop across a contact pair is measured while a known current is driven through only one of the two contacts (by involving a third contact). The measured voltage drop divided by the current yields the contact resistance plus the cable resistance. This is explained in more detail below and in Fig. 2.

Measurements of the contact resistance should be performed under the same conditions as measurements of the QHR for metrological purposes, especially with the same current amplitude. For measurements of a voltage contact, the current should be kept low (typically about 10 μA, a conservative safety limit) to prevent damage to the contact (though the EG-based devices are able to withstand more current flow than GaAs QHRs [29]).

In GaAs QHR devices, the existing guidelines suggest that voltage contact resistances should be kept below approximately 10 Ω (for the *i* = 2 plateau) to avoid significant deviations in the quantized value [2]. For EG-based devices, the situation is similar; however, optimized fabrication techniques such as the use of split or edge contacts can significantly reduce the contact resistance [11, 44]. In well-fabricated graphene QHR devices, typical contact resistance values have been reported to be below 5 Ω (in some cases even in the milliohm or microohm range) [11, 12, 18, 45].

There are several methods for measuring contact resistance. However, a good compromise between complexity and accuracy is the 3-terminal technique mentioned above and schematized in Figure 2. The current is passed through the device on the Hall plateau (*R*_xx_ ≈ 0) between contacts 1 and 2 to identify the contact resistance at contact 1. Assuming zero dissipation, the potential difference between contacts 1 and 7 is accumulated across the series sum of the contact resistances of current contact 1 in which the current is drained, and the lead resistance of the line between contact 1 and the current source low terminal outside the system. The lead resistance must be measured in a separate experiment with the cryostat cold, but this only needs to be done once as lead resistances are not expected to change with time.

For the contact resistance check, there is no particular demand for specialized instrumentation. Satisfactory results can be obtained with commercial current and voltage sources that allow limiting the applied current to about 10 μA, in combination with a voltmeter that provides sufficient resolution for detecting contact resistances of the order of a few ohms or less in series to the lead resistances (which might be as large as 100 Ω).

## 5. Conditions of quantization

### 5.1. Evaluation of Residual Longitudinal Resistivity

While the core tests for ensuring a dissipationless state (*R*_xx_ ≈ 0) remain essential, EG’s unique electronic properties make satisfying these conditions more straightforward than with the GaAs-based devices detailed in the original guide [2]. The key difference with EG is that the condition of a near-zero longitudinal voltage *V*_x_ is achieved over much wider and more accessible ranges of magnetic flux density and temperature [30]. Nevertheless, a determination of the magnetic flux density dependence of the longitudinal and Hall resistances is still required to verify that the quantum Hall effect regime occurs in the expected parameter space (temperature and magnetic flux density), as it can deviate with time in case of drift of the carrier density.

### 5.2. Temperature and Current Dependence

The existing guide [2] dedicates considerable attention to the potential temperature dependence of *R*_xy_ and the methods for applying a correction based on the *s*-parameter (see section 6 below). For EG, such corrections are unnecessary. While *R*_xy_ is generally considered invariant with temperature at the center of the *i* = 2 plateau, this stability should be qualified. For typical EG-based devices, *R*_xy_ remains invariant within metrological uncertainties over a range of 2 K to 5 K [27]. However, this may not hold for devices operating near the plateau boundaries or those with low carrier densities (< 1 × 10^11^ cm^−2^), where temperature-driven transitions can introduce measurable deviations. Graphene’s large Landau-level spacing ensures that *R*_xy_ is invariant over an appreciable temperature range well above temperatures required for GaAs (greater than 10 K) [27].

The check for invariance of *R*_xy_ with respect to the measurement current is still important. However, the higher critical currents in EG-based devices imply that this invariance holds over a much larger range of currents (often up and sometimes beyond 0.5 mA) [12], [14]. This improved range allows for measurements with a better signal-to-noise ratio without inducing dissipation [29].

### 5.3. Magnetic Flux Density Dependence

The QHR in EG-based devices maintains quantization at the *i* = 2 plateau with more robustness along a larger range of magnetic flux densities than in semiconducting heterostructures, and these extraordinarily broad quantum Hall plateaus are understood in the framework of the magnetic-flux density dependent charge transfer from SiC to EG [46, 47]. For epitaxial graphene devices with a carrier density larger than 1 × 10^11^ cm^−2^, the longitudinal resistance (*R*_xx_) and the flatness of the Hall plateau have been verified with a magnetic flux density up to 14 T for metrological purposes [12, 14, 18]. Typically, an acceptable magnetic flux density for metrologically suitable Hall quantization (i.e. nΩ/Ω level quantization of the Hall resistance) is a few tesla higher than the apparent onset of the plateau (where onset may be interpreted as the same magnetic flux density where *R*_xx_ becomes less than 1 Ω) at 4 K or another desired temperature. For instance, the threshold magnetic flux densities of magnetotransport measurements and metrologically suitable Hall resistance quantization are 2 T and 5 T, respectively. In EG-based devices with a carrier density below 1 × 10^11^ cm^−2^, on the other hand, non-monotonic changes of *R*_xx_ and of the Hall quantization have been observed with respect to magnetic flux density [16], which may be due to charge inhomogeneities around the Dirac point in EG.

## 6. Measurement of the QHR

Before a graphene-based device may be used as a primary standard of resistance, it needs to go through a detailed characterization procedure to assess its ideal operating conditions, properties, and device integrity.

### 6.1. Characterization Procedure

Based on the procedure used during the first BIPM.EM-K12 key comparison involving graphene devices [3], the following steps are performed to verify the integrity of EG QHR devices that are going to be used for calibration services as primary resistance standards (and this approach remains generally the same as that for GaAs-based devices):

1. *Overview sweeps:* Measuring the longitudinal resistance and the Hall resistance during continuous magnetic flux density sweeps to roughly identify the flux density at which *R*_xx_ ≈ 0 Ω and *R*_xy_ ≈ *R*_K_/2 (recall that *R*_K_ is the von Klitzing constant). This step does not necessarily require precision equipment and can be done using instruments such as resistance bridges or lock-in amplifiers. If the derived charge carrier density of a graphene-based device is significantly smaller than 1 × 10^11^ cm^−2^, it likely doesn’t provide sufficiently low *R*_xx_ in the μΩ-range and usually exhibits low critical current levels. Thus, such devices are typically excluded from further characterization steps and use as standards (for calibration services).
2. *Contact resistance:* Three-terminal contact resistance measurements should be performed at a fixed magnetic flux density *B* (see Section 4). The value of *B* must be selected from within a range identified from the overview sweeps of step 1, namely where the longitudinal resistance becomes < 1 Ω. The three-terminal resistance includes the cable resistance of one line as well as the remaining longitudinal resistance in the device. After accounting for these resistance components, the actual contact resistances are estimated. All the contacts should be tested, and the use of devices with suspicious, non-uniform contact resistances valued significantly above 10 Ω, or with non-ohmic behaviors should be heavily reconsidered.
3. *High-accuracy characterization of R*_*xx*_ *and the QHR plateau:* To identify the optimal magnetic flux density range for the operation of the EG-based QHR device, the longitudinal resistance is determined at various values of *B*. It is evaluated from the difference between two Hall resistance values, obtained from diagonally and orthogonally aligned contact pairs. These individual Hall resistances are determined using a cryogenic current comparator (CCC) measurement bridge and a 100 Ω reference resistor. An alternative method for measuring *R*_*xx*_ is using a DC current source and a very sensitive nV- or pV-meter, mainly to measure the voltage drop between two contacts on the same side of the device (e.g. between contacts 3 and 5 shown in Figure 2). To minimize offsets, one may apply direct current with reversed polarity. The longitudinal resistance of both sides of the device should be measured and is considered sufficiently low when *R*_xx_ is at the level of 10 μΩ within the combined expanded measurement uncertainty (*k* = 2). The measurement results obtained in this step may also be used to evaluate the *s*-parameter (see next subsection). Note: The value of 10 μΩ is a conservative assignment originating from the requirement to maintain a total QHR uncertainty at the nΩ/Ω level (and assumes an *s*-parameter of about 1).
4. *Uniformity checks at fixed flux density with the CCC involving all contact pairs:* Here, the device integrity is extensively verified at fixed *B* before it is used for calibration services. The suitable *B* value (colloquially known as the “sweet spot”) is identified based on the results in the previous step. The Hall resistances of all available orthogonally aligned contact pairs, as well as of all combinations of diagonally aligned contact pairs, are measured using the CCC measurement bridge and the 100 Ω reference resistor. The integrity of the device is confirmed if the longitudinal resistivities at the high- and low-potential sides of the Hall bar are all on the level of 10 μΩ (within the combined expanded measurement uncertainty (*k* = 2)) and if the Hall resistances determined at all available orthogonally aligned contact pairs match within a few nΩ/Ω.
5. *Evaluation of the correction to be applied to the Hall resistance:* When the QHR is used as a primary resistance standard for calibrations, the *s*-parameter (defined below) value may be used to apply a correction to the value of measured Hall resistance to account for an offset from *R*_K_/2 in the event of a non-zero *R*_xx_. The correction adjusts for the deviation of the measured Hall resistance from *R*_K_/2 and is described by Δ*R*ₓᵧ = *R*_xy_ - *R*_K_/2 = s*ρ_xx_. The uncertainty attached to the correction is described by $u\left(\Delta R_{\text{x}\gamma }\right)=\sqrt{\rho_{\text{xx}}^{2}*u^{2}\left(s\right)+s^{2}*u^{2}\left(\rho_{\text{xx}}\right)}$, where *u*(*s*) and *u*(*ρ*_xx_) are the uncertainties attached to the *s*-parameter (see below) and the longitudinal resistivity. Here, ρ_xx_ is determined in previous steps (where *ρ*_xx_= *R*_xx_ * (*w*/*l*) with *w* and *l* being the width and length of the relevant portion of the device, respectively).

### 6.2. *s*-parameter

The *s*-parameter in the context of QHR metrology is defined as a quantitative measure of the sensitivity of the Hall resistance *R*_*xy*_ to residual dissipation caused by non-zero *R*_*xx*_ in the device. It is a device property that must be determined through measurements if the value of *R*_xx_ is non-zero within the measurement uncertainty. Once *R*_xx_ and the *s*-parameter are known, a correction to the Hall resistance can and must be applied (see step 5 above).

**Figure 3.**
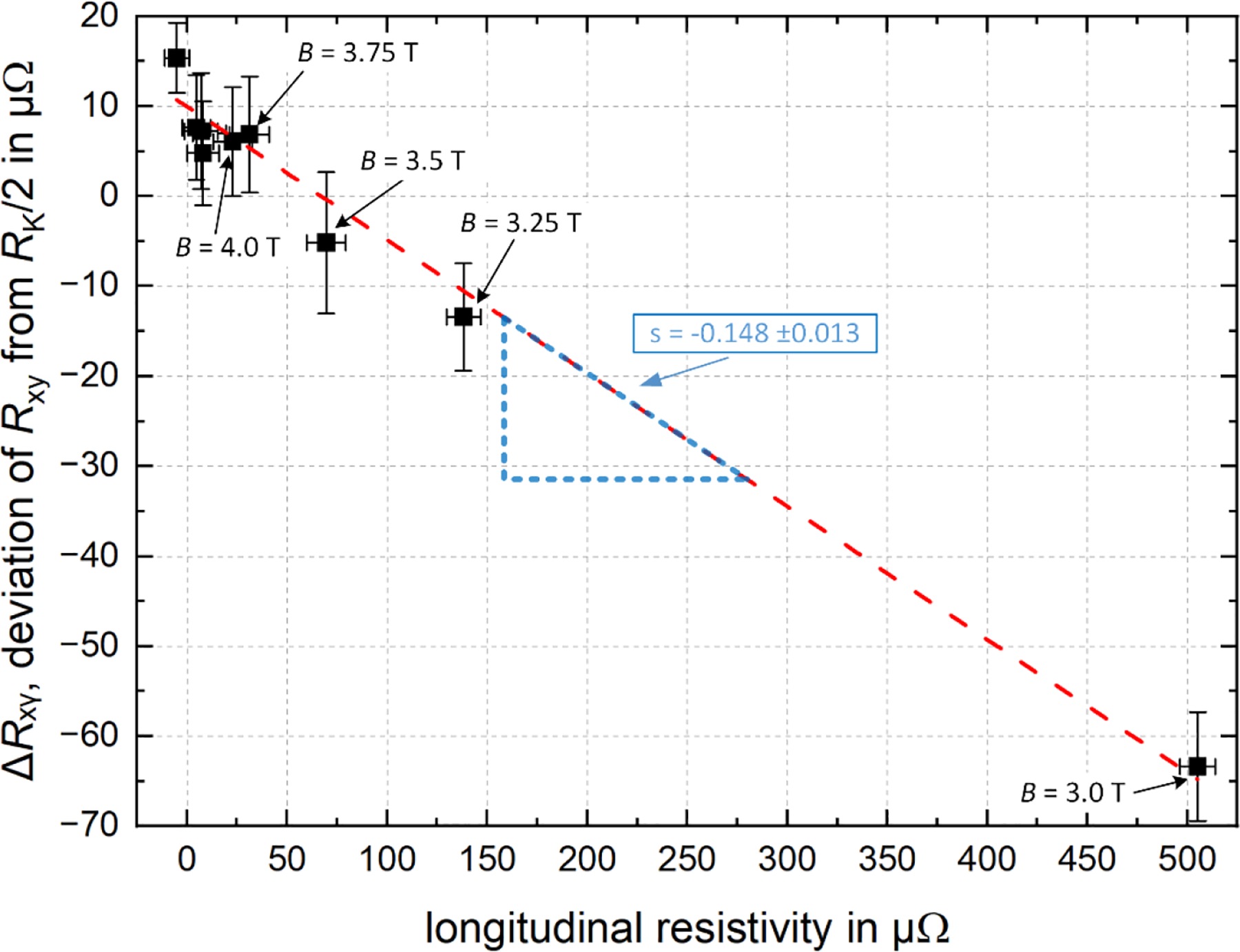
Evaluation of the *s*-parameter from linear regression analysis. The data was obtained from varying the magnetic flux density at the edge of and within the QHR plateau of a graphene QHR device. The *s*-parameter is given by the slope of the linear fit, describing the deviation in the Hall resistance from *R*_K_/2 as a function of *ρ*_xx_.

Since the QHR device tends to change between different cool down cycles, *s* may be determined after each cool down. One common approach is to extract the *s*-parameter value from the linear regression of the Hall resistance deviation versus *ρ*_xx_ (cf. Fig. 3). Experimentally, the *s*-parameter is determined by deliberately varying the operating conditions (e.g., bias current, magnetic flux density, or temperature) and fitting the observed deviation in the quantized Hall resistance from *R*_K_/2 at the *i* = 2 plateau, Δ*R*ₓᵧ = *R*_xy_ - *R*_K_/2, versus *ρ*_xx_ such that the slope, *s*, is represented by *s* = Δ*R*ₓᵧ/Δ*ρ*_xx_ [2].

A smaller *s*-parameter indicates that the device’s Hall resistance is less sensitive to residual dissipative effects. In well-fabricated EG QHR devices, *s*-parameter values have been reported to be typically between ±0.1 and ±0.5 [2], [12], [14], though recent observations of *s*-parameter values of *s* = 2 have been reported [18]. The *s*-parameter values may take on negative or positive values.

## 7. Consistency of QHR measurements for different devices and different quantum numbers

QHRs in two devices (of similar or different composition) can be directly compared for consistency using a probe with dual-sockets [31] or the commonly used (“metrology standard”) TO-8 socket and two-stacked devices [17]. The quantization condition for a particular value of magnetic flux density in both devices can be easily satisfied if one of two devices is made of EG due to the wide Hall plateau. This is generally not the case when the two QHRs include a GaAs/AlGaAs device and Si-MOSFET device. Certainly, QHRs realized in two separate cryomagnet systems can be compared with a bridge based on a CCC or with a direct current comparator (DCC). See Section 9 for more details on using either bridge for precision measurements.

Most accurate consistency checks (uncertainty < 1 nΩ/Ω) are carried out by comparing two QHR devices mounted on the same probe and using a CCC bridge configured in a 1:1 ratio. However, a common operating magnetic flux density value for which both devices are quantized must be available (which is *a priori* the case for comparisons between GaAs- and EG-based devices).

Consistency checks can also be made with a slightly higher uncertainty of about 1 to a few nΩ/Ω with the two devices in two different cryomagnetic systems, and by performing an indirect comparison with a CCC bridge and a transfer resistance standard (conventional resistor). In that case, the optimum operating magnetic flux density can be chosen for each device.

It should be noted that the use of the *i* = 6 plateau is highly uncommon in EG due to inconvenient plateau sizes [48].

## 8. Comments on generic measuring equipment

Compared to GaAs-based devices, EG-based devices can operate at higher temperatures (4 K or more) and lower magnetic flux densities (around 5 T or less), due to the larger Landau level spacing in graphene, without significantly increasing the longitudinal resistivity. This facilitates the use of compact, dry cryostats [29, 35]. EG-based devices also have the advantage that they can be employed with significantly higher measurement currents up to hundreds of μA for single devices or even mA in array configurations [15].

### 8.1. Cryogenic and Non-Cryogenic Systems

Traditionally, liquid-helium based cryostats with a superconducting magnet are used. These cryostats have the advantage of offering a quiet environment in terms of vibrations and electromagnetic pollution, together with adequate thermalization of the measuring cables as this could be done directly through the liquid helium, at 4.2 K. However, because of the elevated costs and scarcity of liquid helium, a shift towards the use of cryogen-free systems is progressing in quantum electrical metrology. While these systems are globally easier to use than the liquid-helium based counterparts, have a lower maintenance cost and do not require expertise in cryogenics to be used, they suffer from a higher vibration and electromagnetic perturbation level. In addition, because there is no liquid helium, the thermalization of the cables and samples becomes critical. All in all, these can affect quantum Hall measurements and hinder high-precision measurements. Quantum Hall measurements in such systems have been demonstrated [29, 35, 49, 50, 51], but several precautions have to be taken, due to increased sensitivity to vibrations, electromagnetic interference, and temperature fluctuations [18, 29, 35, 51]:

1. Mechanical vibration isolation – Pulse-tube cryocoolers, common in dry cryostats, introduce mechanical vibrations that can lead to microphonic noise in the measurement wires and local Joule heating. Such vibrations can disturb the delicate quantization of the Hall resistance. Therefore, effective mechanical decoupling and damping strategies are essential to minimize these disturbances [49, 50].
2. Temperature stability and thermal anchoring – Precise quantization requires not only low temperatures (typically in the 2 K to 5 K range for graphene) but also excellent temperature stability. The thermal contact between the sample and the cryostat cold head must be optimized to reduce temperature gradients and fluctuations, which could lead to a finite longitudinal resistivity and deviations from ideal quantization.
3. Electromagnetic interference – Electromagnetic interference (EMI) from the cryocooler’s electronics may introduce noise [16, 29, 49, 51]. Thus, careful design of RF shielding, proper coaxial cabling, and grounding strategies are imperative to minimize extraneous signals that could perturb the measurement of the quantized Hall resistance.

### 8.2. Mechanical Vibration Isolation

Cryocoolers inherently produce vibrations at the vicinity of the cold finger, mainly from the valve unit. If possible, it should be separated from the cold head, as a remote motor, and solidly anchored and independent from the cryostat [49, 50, 51]. However, it seems extremely difficult to damp the vibrations to their full extent, and some can still be transmitted to the measuring insert [51].

Overall, the effects of dry cryostat mechanical noise on precision QHE measurements, and possible mitigation strategies, are considered in [29, 35, 49, 50, 51]. Chae *et al*. [49,50] and Taupin *et al*. [51] discuss in detail the effect of mechanical vibrations on the probe voltage and current noise spectra, and on the Allan deviation of both DCC and CCC measurements. Reference [50] analyzes the effectiveness of a simple vibration damper between the compressor and the cryostat, whereas Ref. [51] provides help in quantifying the mechanical noise and the relationship with the CCC superconducting quantum interference device’s (SQUID) detector noise spectrum.

### 8.3. Temperature Stability and Thermal Anchoring

The thermalization and temperature stability in a cryocooler can be problematic as, without particular precautions, the sample is under vacuum, and the second (low temperature) stage of a cryocooler tends to exhibit significant temperature fluctuations (on the order of a few tenths of a kelvin at 3 K). For measurements under these circumstances, dampening the temperature variation of the sample holder stage is essential and thermalization of the cables at the first and second stages is necessary, which can be tedious if using coaxial cables for measurements in the AC regime.

One solution is to use a system with a variable temperature insert (VTI), in which the sample holder is a continuous helium flow, not directly in contact with the second stage. With such a system, the sample and cables are immersed in exchange gas, facilitating the thermalization, and the lowest accessible temperatures may reach 1.3 K with superior stability (better than 1 mK over several hours) [51]. Note that the temperature instability at the sample holder can be as small as a few mK if the helium exchange gas is introduced into the sample space in contact with a liquid helium pot in the second stage of a cryocooler in a top-loader system [50].

### 8.4. Electromagnetic Interference

Since quantum Hall devices require magnetic flux, residual vibrations in the system easily induce electromagnetic interference, giving rise to signal noise. The intrinsic QHR noise level from vibrations is, thus, expected to be much larger in a cryocooler (under field) than in a liquid-helium based cryostat because of the remaining vibrations. The effects of magnetic field noise (Type A uncertainty components) on the measurement uncertainty may be reduced by decreasing magnetic field amplitude [51].

Precautions must be taken to prevent interferences that can detrimentally affect the measurements. Observations at NMIs have been made suggesting that perturbations at high frequency (several 100 kHz) are directly caused by the cryocooler. Consequently, instabilities of a direct current (DC) SQUID when incorporated in a CCC have been observed, in part because the modulation signal of the DC-SQUID is situated at a frequency within the perturbation frequency band. These instabilities are not apparent when using a RF-SQUID with a much higher modulation frequency (around 19 MHz) [51]. A careful choice of instrumentation is essential.

## 9. Precision resistance measurement bridges

### 9.1. CCC Resistance Bridges

The CCC bridge is a bridge based on a quantum current comparator that allows for highly accurate comparison of resistance standards. It is commonly used by NMIs and the BIPM to transfer the QHR value to conventional resistance standards with lowest achievable uncertainty.

**Figure 4.**
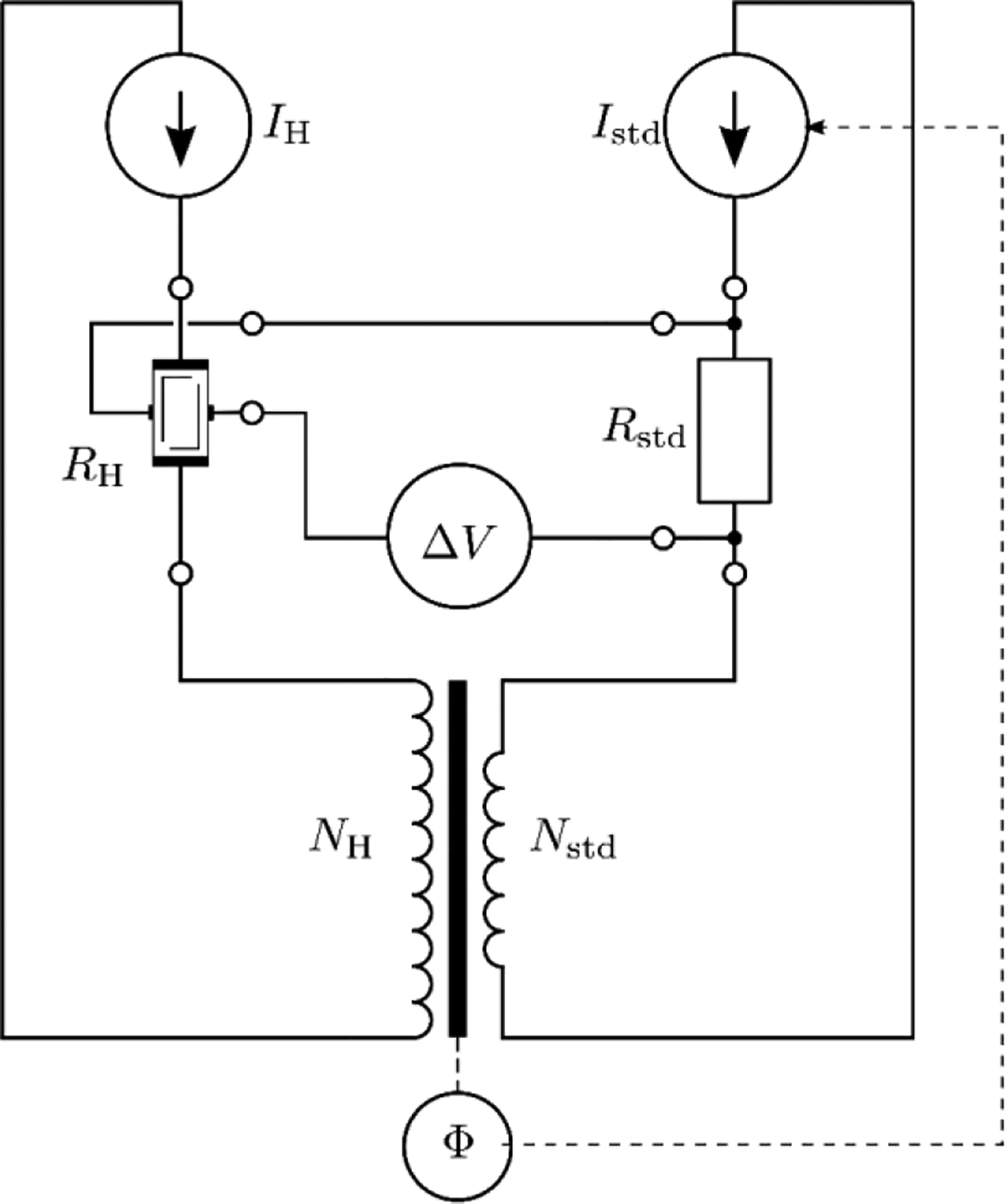
A simplified schematic diagram of a current comparator resistance bridge (CCC or DCC bridge).

Figure 4 shows a functional diagram of CCC bridge, which also applies to DCC bridges (see details next sub-section). In this figure, the quantum Hall reference resistor *R*_H_ and the artifact (device-under-test) resistor *R*_std_ are defined as four-terminal standards. The comparator has two isolated current sources which generate *I*_H_ and *I*_std_. The currents flow through *R*_H_ and *R*_std_, and also through the windings of the current comparator having turns *N*_H_ and *N*_std_. An active feedback loop maintains (nulls) the flux *Φ* in the comparator, and hence the relation *N*_H_ *I*_H_ + *N*_std_ *I*_std_ = 0.

The residual voltage difference Δ*V*, measured by a nanovoltmeter, is the input quantity of a measurement model that provides the reading *Q* = *R*_H_ / *R*_std_. In a CCC, the magnetic flux is constrained by superconducting shields, and the flux detector is a superconducting quantum interference device with a pickup winding coil. In a DCC, the flux path is given by a ferromagnetic core; a modulation scheme (fluxgate detection) is employed to sense the residual DC flux.

Commercially available CCC resistance bridges provide adjustable winding ratios that enable measurements with a large variety of resistance ratios with high accuracy, cf. Table 3.

### 9.2. Room-Temperature Resistance Bridges

The robustness of the QHE in EG and its relaxed operation conditions make the direct measurements with room-temperature resistance ratio bridges particularly attractive. However, even in best cases, the measurement uncertainties achievable with these bridges typically are at least one order of magnitude higher compared to best measurements performed with CCC-based bridges.

Precision resistance ratio bridges are commonly employed at NMIs and calibration centers for comparing artifact resistance standards and maintaining resistance scales. Nowadays, the most accurate room-temperature ratio bridges are those based on the direct current comparator (DCC) principle. The measurement accuracy is limited by the sensitivity of the magnetic flux detector and of the voltage detector, and by the magnetic flux leakage occurring in the DCC core. DCC bridges are versatile, capable of measuring resistances ranging from the μΩ to the MΩ range. They achieve their best accuracy when measuring medium-ranged resistors, specifically those within the range of 1 Ω to 10 kΩ, and when the measured resistance ratio is in the range of 0.1 to 10, or slightly beyond.

**Figure 5.**
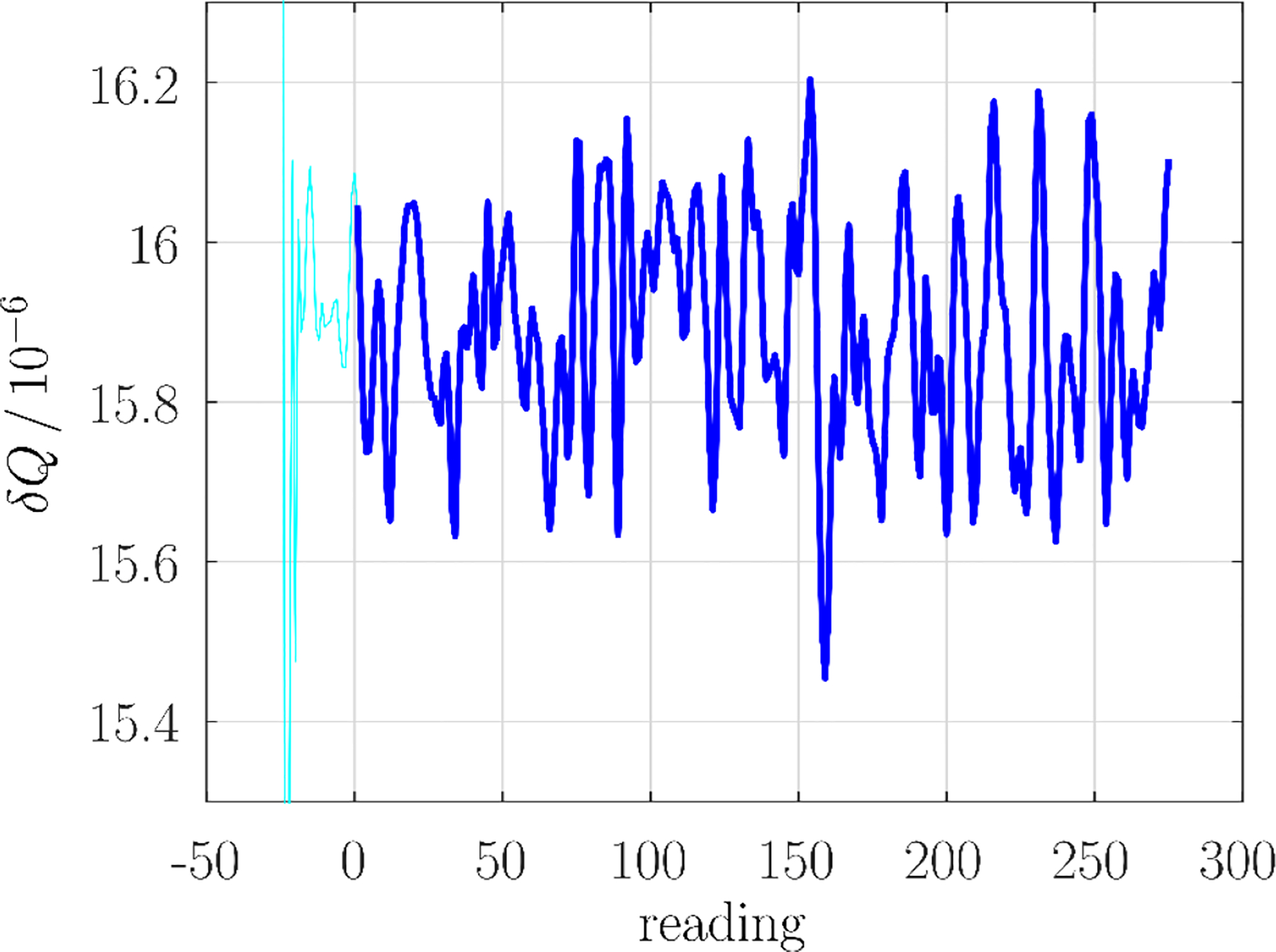
Typical outcome of a measurement performed with an automated DCC bridge, comparing the ratio between the QHR of a graphene-based device and a thermally stable 1 kΩ standard resistor. The exemplary EG-based device is measured in a dry cryostat with a magnetic flux density of 5.5 T and with an applied current of 50 μA. The plot shows the time series of the relative deviation δ*Q* from the nominal ratio *R*_H_ / 1 kΩ. The initial readings (light blue) show some instability and are discarded from subsequent data analyses. The overall measurement time is about 4000 s. The readings are correlated in time: a statistical analysis shows a correlation length of about 32 points, which must be considered to evaluate the type A contribution to measurement uncertainty. Measurements performed on a room-temperature resistor of nominal value *R*_H_ show similar noise levels, confirming that the overall measurement noise is dominated by the sensitivity of the DCC and not significantly increased by noise coupling to the dry cryogenic environment.

In performing measurements with DCC bridges on EG-based devices, the following should be considered:

1. A DCC bridge is less sensitive to environmental noise than a CCC bridge – the use of a dry cryostat, which has electromagnetic noise generated by its mechanical vibrations, is therefore less critical.
2. DCC bridges can be asymmetrical: the total number of turns selectable for the two principal windings might differ. When measuring resistance ratios not close to unity, the resistor of larger magnitude must be connected to the winding having the larger number of turns to maximize the flux detector sensitivity.
3. The DCC bridge ratio can be calibrated [52, 53], and the calibration is stable over time [52, Fig. 3], so the bridge ratio error can be corrected to a certain extent. The error can be dependent on the specific settings of the bridge, in particular the measurement current, or on the deviation from the nominal ratio of the resistors (i.e. the nV reading of the DCC bridge) [53, Fig. 10]. This is particularly relevant when performing test measurements at different current levels, for example, to identify the operating current range of the device or when the load coefficient of an artifact resistor must be determined.
4. Automated DCC bridges typically perform measurements in two stages. A coarse measurement is carried out to identify the proper turn ratio setting; then the precision measurement sequence starts. In some bridges, current glitches occurring during the rough phase have been observed. The glitches are irrelevant in a measurement of a normal artifact standard but might drive (possibly irreversibly) the QHR out of the quantization regime.
5. Automated instruments perform automatic current reversals and display a running average; in addition, some instruments perform an internal digital filtering of the readings. This induces autocorrelation in the time series of readings being acquired [53, Figs. 4–5]. If this occurs, the standard deviation of the mean is no longer a correct estimate of the Type A uncertainty of the measurement, and better estimators must be considered. Among methods of evaluation of the Type A uncertainty there are the Allan deviation and the autocorrelation estimation, as proposed in ISO 21485:2022 [54].
6. Like CCC bridges, DCC bridges can measure *R*_xx_ by using the “diagonal method” (see Section 6), but with a lower sensitivity. Some bridges offer a special mode that allows performing a direct *R*_xx_ measurement, and for related Kelvin bridges, EG-based devices have shown compatibility [55].

### 9.3. Low-Frequency AC Bridges

Similar in its operating principle to the DCC bridge (Fig. 4), the low-frequency AC bridge developed by F. Delahaye is another room-temperature alternative technique to measure resistance ratios [56], including QHR to conventional resistance standards. The advantage of using ac rather than dc is the improved rejection of thermal emf and other low-frequency noises.

This type of bridge is based on a room-temperature current comparator operating with low-frequency sinusoidal signals (typically 1 Hz to 20 Hz), often referred to as a low-frequency current comparator (LFCC) [57]. As for the DCC, the primary and secondary windings of a LFCC are wounded on the same high-permeability toroidal core and generate opposite magnetic fluxes whose cancellation is a prerequisite for bridge balancing. The zero-flux detection is made using a third winding of high number of turns whose inductance is coupled with an additional parallel capacitance that can be tuned to adjust its resonance frequency to the excitation frequency of the bridge.

The performance of an LFCC is based on the use of electrostatic and magnetic shields to prevent any coupling between the detection winding and the primary and secondary windings, and on a specific coaxial guarding circuit of the primary and secondary windings. The precision level of resistance ratio measurements performed with LFCC-based bridges can be as low as nΩ/Ω. However, the specific guarding scheme of the LFCC windings depends on its ratio, implying that a different LFCC is required for each ratio value measured, making LFCC-based bridges less versatile than DCC bridges. For example, measuring the *R*_H_(2)/100 Ω ratio will require an LFCC with a ratio of 129.06/1 (e.g., winding ratio 2065/16), and scaling from 100 Ω to 1 Ω or 10 kΩ will require an LFCC with a ratio of 100/1 (e.g., winding ratio 1500/15). Any other ratio, decimal or non-decimal may be considered, provided that the practical constraints associated with the fabrication of the LFCC can be overcome (number of turns, size, parasitic impedances, etc. [56]).

Furthermore, as LFCC bridges do not measure dc ratios, an ac-dc correction must be applied to the measured ac ratio. This correction includes both the ratio error of the bridge, and the frequency dependence of the resistance ratio measured. It can be determined by comparing measurements of this ratio carried out with the LFCC bridge and a CCC bridge.

For dc measurements, LFCC bridges are then referenced to a CCC bridge. The BIPM has been using LFCC bridges since the 1990s for routine calibration and key comparisons of QHR systems with an uncertainty level of typically 2 nΩ/Ω (BIPM.EM-K12 ongoing on-site comparison program [3]). The same level of uncertainty can be achieved for resistance ratio measurements at 1 Hz with a state-of-the-art LFCC and bridge electronics (dual current source, feedback circuit and current compensation network).

## 10. Conclusion

In summary, EG-based devices represent a transformative advancement in electrical metrology, surpassing traditional GaAs heterostructures by offering highly stable and scalable access to the QHR under significantly relaxed operating conditions [58]. And despite EG’s surface-exposed nature necessitating sophisticated doping and encapsulation strategies, the resulting devices exhibit robust, dissipationless transport and minimal contact resistance through optimized Hall bar geometries and multiple-series connections.

To fully leverage advantages from EG-based devices, laboratories are advised to follow a rigorous, five-step characterization protocol. This process begins with comprehensive magnetic flux density sweeps to locate the quantized plateau and is followed immediately by a dedicated check of contact resistances. Because low contact resistance is a major precaution against heating and other device errors, verifying that these values remain within acceptable limits is a prerequisite for subsequent steps. Once the contact resistance condition is met, a high-accuracy determination of the longitudinal resistance must be performed to identify the region of the plateau where dissipation is minimized. This is complemented by uniformity checks across various contact pairs to ensure the Hall resistance is consistent throughout the device. The final stage of this procedure involves a formal evaluation of the *s*-parameter. Ultimately, EG-based resistance standards help streamline the characterization process and provide a more versatile, high-precision primary resistance standard for modern cryogenic environments.

## Figures and Tables

**Table 1.** Example parameters reported for tested EG-based QHR devices, including the range of carrier density, critical current, and *s*-parameter. This is not an exhaustive list, but rather, demonstrates typical observed values.

Fabrication source	Range of carrier density	Critical current†	*s*-parameter	Ref.
Physikalisch-Technische Bundesanstalt (PTB)	≈ 1.0 × 10^11^ cm^−2^ to 3.0 × 10^11^ cm^−2^ (*n*- or *p*-type)	≥ 320 μA (*R*_*xx*_ ≤ 10μΩ*)	typ. between ±0.1 and ±0.5	[12], [13], [14], [2]
Research institutes of Sweden (RISE)	1.3 × 10^11^ cm^−2^ to 1.7 × 10^11^ cm^−2^ (*n*-type)	≈ 100 μA, 5 T, 4 K	Not reported^#^	[15, 38]
National Institute of Standards and Technology (NIST)	0.7 × 10^11^ cm^−2^ to 1.0 × 10^12^ cm^−2^ (*n*-type)	*ρ*_xx_ ≈ 47 μΩ @ 77.5 μA and *B* = 5 T	Not reported^#^	[29]
Korea Research Institute of Standards and Science (KRISS)	≈ 2.3 × 10^11^ cm^−2^ to 3.4 × 10^11^ cm^−2^ (*p*-type)	≥ 40 μA (6 T)	−1.1 to −0.47	[16], [17]
Laboratoire national de métrologie et d'essais (LNE)	0.5 × 10^11^ cm^−2^ to 5 × 10^11^ cm^−2^	≈ 350 μA	≤ 2	[18]

† Criterion for decision: When *R*_*xy*_ goes above 10 nΩ/Ω from the calculated value, plateau may be considered not quantized.

* Electrical current limited by cryogenic current comparator (CCC) system.

# Data may be part of ongoing experiments.

**Table 2.** Consolidated summary of practical EG operating conditions.

Measurement Parameters	Ranges of Operation
Temperature	≈ 1.0 × 10^11^ cm^−2^ to 3.0 × 10^11^ cm^−2^ (*n*- or *p*-type)
Magnetic Flux Density	1.3 × 10^11^ cm^−2^ to 1.7 × 10^11^ cm^−2^ (*n*-type)
Electrical Current	0.7 × 10^11^ cm^−2^ to 1.0 × 10^12^ cm^−2^ (*n*-type)
Carrier Density	≈ 2.3 × 10^11^ cm^−2^ to 3.4 × 10^11^ cm^−2^ (*p*-type)

* Upper bound will vary based on device configuration.

**Table 3.** Typical measurement parameters and achievable uncertainties of commercially available CCC resistance bridges. Data taken from BIPM.EM-K12 on-site comparison [3].

Measurement parameters	Resistance ratio		
	QHR_*graphene*_ / 100 Ω	10 kΩ / 100 Ω	QHR_*graphene*_ / QHR_*GaAs*_
Winding ratio *N*_1_/*N*_2_	4001/31	4100/41	4096/4096
Peak-to-peak voltage across resistor / V	1	1	1
Typical duration of individual measurement	20 min	25 min	30 min
Typical type A standard uncertainty (*k* = 1) for duration of individual measurement / 10^−9^	0.180	0.694	0.179
Typical type B standard uncertainty (*k* = 1) / 10^−9^	0.094	0.060	0.118

## Key Terms:

- hot spot - A region of localized heating and high current density that occurs at the source and drain contacts of a graphene-based device. In the quantum Hall regime, current enters or exits the device predominantly at a specific corner of the electrical contact, leading to a concentrated area of energy dissipation.
- edge channel - One-dimensional conducting paths located at the physical boundaries of the graphene sample. In the quantum Hall regime, the bulk of the material remains insulating, while charge transport is restricted to chiral quantum edge states that flow without backscattering.
- Landau level - Discrete, highly degenerate energy levels formed by the quantization of the circular orbits (cyclotron motion in the semi-classical picture of physics) of charge carriers in a two-dimensional system when subjected to a perpendicular magnetic field.
- filling factor - A dimensionless quantity representing the number of occupied Landau levels.
- equilibration length - The characteristic distance over which charge carriers in an edge channel reach a steady state.
- dissipationless transport - A transport regime where longitudinal resistance vanishes, meaning current flows through the device without energy loss or resistive heating.

## List of Abbreviations:

QHR: Quantized Hall Resistance
QHE: Quantum Hall Effect
EG: Epitaxial Graphene
NMI: National Metrology Institute
CCC: Cryogenic Current Comparator
DCC: Direct Current Comparator
LFCC: Low-Frequency Current Comparator
BIPM: Bureau International des Poids et Mesures
CCEM: Consultative Committee for Electricity and Magnetism
ZLG: Zero-Layer Graphene
VTI: Variable Temperature Insert
EMI: Electromagnetic Interference
ESD: Electrostatic Discharge
SQUID: Superconducting Quantum Interference Device
MOSFET: Metal-Oxide-Semiconductor Field-Effect Transistor
PASG: Polymer-Assisted Sublimation Growth
